# Sym3DNet: Symmetric 3D Prior Network for Single-View 3D Reconstruction

**DOI:** 10.3390/s22020518

**Published:** 2022-01-11

**Authors:** Ashraf Siddique, Seungkyu Lee

**Affiliations:** Department of Computer Science and Engineering, Kyung Hee University, Giheung-gu, Yongin-si 17104, Gyeonggi-do, Korea; siddique2127@khu.ac.kr

**Keywords:** 3D object reconstruction, reflection symmetry, deep learning

## Abstract

The three-dimensional (3D) symmetry shape plays a critical role in the reconstruction and recognition of 3D objects under occlusion or partial viewpoint observation. Symmetry structure prior is particularly useful in recovering missing or unseen parts of an object. In this work, we propose Sym3DNet for single-view 3D reconstruction, which employs a three-dimensional reflection symmetry structure prior of an object. More specifically, Sym3DNet includes 2D-to-3D encoder-decoder networks followed by a symmetry fusion step and multi-level perceptual loss. The symmetry fusion step builds flipped and overlapped 3D shapes that are fed to a 3D shape encoder to calculate the multi-level perceptual loss. Perceptual loss calculated in different feature spaces counts on not only voxel-wise shape symmetry but also on the overall global symmetry shape of an object. Experimental evaluations are conducted on both large-scale synthetic 3D data (ShapeNet) and real-world 3D data (Pix3D). The proposed method outperforms state-of-the-art approaches in terms of efficiency and accuracy on both synthetic and real-world datasets. To demonstrate the generalization ability of our approach, we conduct an experiment with unseen category samples of ShapeNet, exhibiting promising reconstruction results as well.

## 1. Introduction

Humans are able to predict the three-dimensional geometry of an object from a single 2D image based on the prior knowledge of target objects and related environments. However, it is quite a challenging task for machines without such prior knowledge because of the drastic variation of object shape, texture, lighting conditions, environments and occlusions. In traditional 3D reconstruction approaches, such as Structure from Motion (SfM) [1] and Simultaneous Localization and Mapping (SLAM), visual appearance consistency across multiple views are utilized to infer lost three-dimensional information, finding multi-view corresponding point pairs. Extracting dense corresponding point pairs is not a trivial task due to texture-less regions, large differences in viewpoints, and self-occlusion [2]. A complete 3D shape can be observed as long as the multiple images cover the target object from entire angles of view. A 3D reconstruction from 2D images is a challenging task that has been studied for a long time. Traditional approaches, such as structure from motion (SfM) and simultaneous localization and mapping (SLAM), require multiple RGB images of the same target scene [3,4]. To recover the 3D structure, dense features are extracted and matched to perform the minimization of re-projection errors [5]. However, detecting corresponding pairs is a difficult task when the distances between multiple viewpoints are large. Furthermore, scanning the entire aspects of an object with 2D color images is computationally expensive especially with occluded or concave surface regions [3]. Taking the advantage of the availability of large-scale synthetic data, deep learning-based networks have been introduced to reconstruct 3D shapes from single or multiple view RGB images. LSM [6] and 3D-R2N2 [7] propose RNN based networks to predict voxel representation of 3D shape from single or multiple view images where input views are processed sequentially using a shared encoder. Extracted features from views are refined incrementally when more input views are available. In their methods, the information from earlier input views is hardly passed to the final refined features for 3D reconstruction. DeepMVS [8] and RayNet [9] apply max and average polling, respectively, over features of entire viewpoints to accumulate outstanding features from unordered multiple views. While these methods alleviate the limitation of RNN, they lose useful view-specific information. Recently, for more effective accumulation of entire viewpoint features, AttSets [10] uses an attentional aggregation module that predicts weight matrix for the features of attention. However, feature aggregation from multiple views is a difficult task in latent space. To overcome the difficulties of feature aggregation, Pix2Vox++ [11] proposes a multi-scale context-aware fusion module that applies context information across multiple view images. In the method, they apply the context-aware fusion module after coarse volume prediction and finally refine the 3D reconstruction using a 3D auto-encoder. However, all these methods learn shape priors implicitly and are sensitive to noisy input or heavy occlusion frequently existing in real-world images. Recently deep learning methods are able to recover 3D shapes in the form of meshes and point clouds. Reference [12] predicts dense point cloud using 2D convolutional operation from multiple viewpoint images and performs geometric reasoning with 2D projection optimization. Pixel2Mesh++ [13] uses a graph convolutional network to recover 3D mesh of objects from cross-view information.

Different from multi-view 3D reconstruction, single view reconstruction has to predict a complete 3D shape based on single view observation and corresponding prior knowledge. Therefore, complete 3D shape reconstruction from a single 2D image is a challenging task. To resolve the problem, several methods have attempted to reconstruct a 3D shape from a 2D view, where the 2D view is either silhouettes [14], shading [15,16], or texture [17]. In real-world scenarios, these methods are not practical for shape reconstruction because of presumption step on camera calibration, smooth surface, single light source, and so forth [18]. To address the issues, most of the state-of-the-art methods map input images to latent representation and obtain the corresponding 3D shape by decoding the latent representation. A joint 2D–3D embedding network proposed in [19], named TL-Network, extracts good latent feature representation using a 3D auto-encoder. Latent features are inferred from 2D input images. But the method reconstructs very low resolution of the 3D shape. With great achievements of generative adversarial networks (GANs) [20] and variational auto-encoders (VAEs) [21], 3DVAE-GAN [22] uses GAN and VAE to reconstruct a 3D shape from a single 2D image. MarrNet [23] and ShapeHD [24] use an intermediate step of estimating silhouettes, depth, and normal of 2D images and reconstructs 3D shape from the depth and normal. To reconstruct a high-resolution 3D shape, OGN [25] proposes octree based learning representation. Matryoshka networks [26] use nested shape layers by recursively decomposing a 3D shape. Currently, several methods are trying to reconstruct a 3D shape with different representations such as point cloud [27], mesh [28] and signed distance field [29]. PSG [27] proposes a method to retrieve point cloud using a simple deep neural network from a single 2D image. Pixel2Mesh [28] reconstructs triangular meshes of 3D shape. Taking the advantage of fine-grained 3D part annotations [30], several approaches [30,31,32] reconstruct 3D shapes by combining 3D parts in a hierarchical scheme.

Reflection symmetry is one of the useful characteristics in both real-world and man-made objects for better description, understanding, and sometimes also being visually attractive. Reference [33] shows the importance of symmetry and virtual views on 3D object recognition. Reflection symmetry has been employed in diverse tasks in computer vision such as novel view synthesis [34], texture inpainting [35] and shape recovering [36]. Symmetry correspondences are utilized for 3D reconstruction with different representation such as curves [37], points [38] and deep implicit fields [39]. To find the symmetry correspondence, these methods need either camera pose or symmetry plane as given inputs. References [40,41] proposes symmetry detection methods for 3D reconstruction. On the other hand, reference [39] propose an implicit function for 3D reconstruction and find the local details by projecting 3D points onto 2D image and applying symmetry fusion. Symmetry prior in considerable number of object types is one of the powerful clues to infer a complete 3D shape from single-view images [42]. Symmetry properties have provided additional knowledge about 3D structures and the entire geometry of objects in 3D reconstruction tasks [43,44,45,46].

In this work, we propose Sym3DNet for single-view 3D reconstruction. Sym3DNet recovers occluded and unseen symmetry shapes by sharing the symmetry feature inside the networks performing 3D reconstruction in a complex environment. Additionally, we incorporate 3D shape perceptual loss that improves the reconstruction of a realistic 3D structure in both global and local 3D shapes. Extensive quantitative and qualitative evaluations on ShapeNet and Pix3D datasets show an outstanding performance of the proposed 3D reconstruction from single image.

## 2. Proposed Method

**Figure 1:**
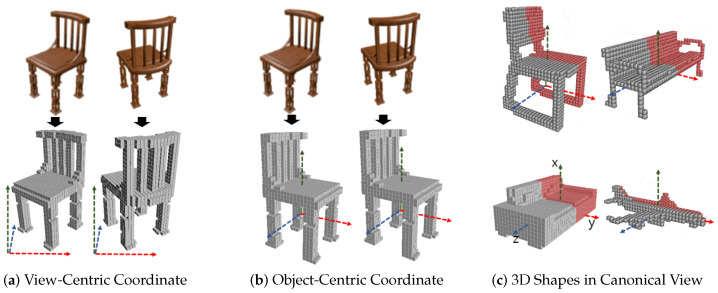
(**a**) View-centric coordinate where 3D shapes are aligned to input image view in 3D camera space. (**b**) Object-centric coordinate where 3D shapes are aligned to common canonical view regardless of views of input images. (**c**) In canonical view, all 3D shapes are aligned in world 3D space. Objects are symmetric with respect to the x-y plane (symmetry plane).

To apply symmetry prior to 3D reconstruction, a view-orientation of 3D output with respect to input view is critical. The orientation of reconstructed 3D output in view-centric coordinate is aligned to the 3D camera space of input image as shown in Figure 1a. On the other hand, in object-centric coordinates, the orientation of the 3D output is aligned to the canonical view as shown in Figure 1b. In the canonical view, orientations of all 3D shapes are aligned in 3D space as shown in Figure 1c. In order to include symmetric prior inside a network, we have to know the symmetry correspondence of the 3D output. To find the symmetry correspondences in view-centric coordinates, symmetry plane detection is essential [41]. In a view-centric coordinate, the global reflection symmetry plane of an object comes to vary along with the change of input view. NeRD [40] has proposed a learning-based reflection symmetry plane detection from a single-view shape image. This method can be extended to pose estimation, depth estimation and 3D reconstruction task. It performs well with synthetic clean object images with uniform backgrounds. However, symmetry plane detection with real-world images with arbitrary backgrounds becomes a more challenging task. Therefore, incorporating an additional step of symmetry plane detection makes the performance of 3D reconstruction dependent on the performance of symmetry plane detection. Global reflection symmetry plane of 3D shapes in object-centric coordinates can be defined in the canonical view. Ladybird [39] has proposed a deep implicit field-based 3D reconstruction where local image features of the corresponding symmetry points are fused to solve the problem of self-occlusion. To extract the local feature from an input image, camera extrinsic parameters are either given or predicted from the input view, which restricts the scope of single-view 3D reconstruction application. The prediction of camera parameters is often very difficult for real-world images and reconstruction performance is affected by the prediction performance of camera parameters.

To exploit the advantage of symmetry in view-centric coordinates, most of the methods have to solve the symmetry detection problem which itself is challenging with real-world images due to self-occlusion or occlusions by other objects and complicated background. On the other hand, other methods using 3D projection onto 2D to collect symmetry correspondence have to know the camera pose which is another challenging task with occlusions and complicated background. In this work, we utilize the advantage of the canonical view and propose voxel-wise symmetry fusion inside our network to share the symmetry prior. Symmetry fusion helps the network to recover the missing shapes in 3D reconstruction.

Instead of using camera information or a separate symmetry detection step [39,40], we focus on the canonical view 3D reconstruction network in which the global symmetry plane of 3D shapes are learned implicitly and aligned, exploiting the benefits of symmetry properties of 3D objects in 3D reconstruction. We propose a Symmetric 3D Prior Network (Sym3DNet) for single-view 3D reconstruction. Sym3DNet is composed of two jointly trained encoder branches: a 3D shape encoder and a 2D image encoder. A 3D decoder is connected to the encoders so that the input 3D volume and 2D image are represented in the same latent space and reconstruct 3D volume from the latent representation via 3D decoder. To incorporate the symmetric prior, the symmetry fusion module is attached to the 3D decoder and perceptual loss is calculated. A pair of symmetry voxels share their receptive features in the symmetry fusion module. To penalize the non-symmetric shape in our network, we introduce a perceptual loss that has two terms: global and local. We pass the reconstructed 3D shape and corresponding ground truth to the pre-trained 3D shape encoder and calculate global perceptual loss based on the latent representation of predicted and original 3D shape. In this way, global perceptual loss tries to penalize not only reconstructed shapes deviating from original shapes but also the prediction of perceptually unpleasant (i.e., visual discomfort) shapes. Many encoder–decoder networks [6,7,10,11] suffer from limited performance in the reconstruction of local details. To recover local details, the local perceptual loss is calculated from the randomly chosen partial 3D data of reconstructed 3D object and their ground truths. For the local perceptual loss, we use a 3D shape encoder pre-trained with randomly cropped small 3D shapes. In our experimental evaluation, the proposed Sym3DNet achieves an outstanding performance of single-view 3D reconstruction in terms of both efficiency and accuracy on ShapeNet [47], a synthetic dataset and Pix3D [48], a real-world dataset. The proposed method is also generalized to unseen shape reconstruction.

The goal of our method is to predict a voxelized 3D shape from a single 2D image. The output of the 3D shape prediction is represented by a 3D voxel grid where 1 indicates the surface of the shape and 0 indicates another empty space. Our method maps the input RGB image to a latent representation of a 3D shape and decodes the representation obtaining the corresponding volumetric shape. The proposed method optimizes the latent space representation of a 3D shape so that the original 3D shape can be reconstructed in the 3D decoder. At the same time, the latent space representation of the 3D shape has to be obtained from the corresponding 2D color image in the 2D image encoder.

**Figure 2:**
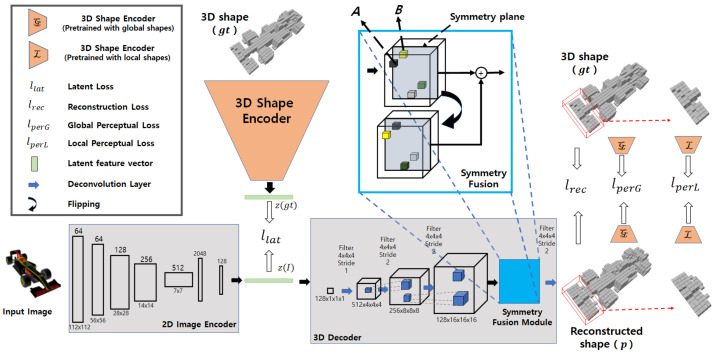
Proposed symmetry 3D prior network: During first stage of our training, the 3D shape encoder and 3D decoder are trained to learn latent space representation of 3D shapes. In second stage, the 2D image Encoder is trained with a pre-trained 3D decoder to learn the mapping between 2D and 3D representations in latent space. Finally, perceptual loss is applied together with latent loss and reconstruction loss.

First, 3D shape encoder and decoder shown in Figure 2 are trained as a 3D auto-encoder. The 3D shape encoder takes the 3D voxel representation gt as input and transforms 3D shape into latent feature vector z(gt). The 3D decoder takes z(gt) as input and decodes the latent feature vector to a voxel representation of a 3D shape. Then, to ensure the transformation of the same latent feature z(gt) from a respective 2D image, our network contains a 2D image encoder shown in Figure 2. The 2D image encoder of our network takes a 2D RGB image *I* as input and transforms the 2D image into latent features z(I) and tries to map with corresponding z(gt) in the second stage of training. Finally, we fine tune the 2D image encoder and the 3D decoder with perceptual and reconstruction losses.

The proposed Sym3DNet consists of two jointly trained encoders: a 2D image encoder and a 3D shape encoder. In particular, two encoders map a 3D shape and corresponding 2D image into the same point of latent space. The 3D output is generated by the 3D decoder from the latent space. As our output is in canonical view, we apply a symmetry fusion module in the 3D decoder to provide the symmetry feature fusion to our model as shown in Figure 2. The symmetry feature fusion module shares the structural shape information in their corresponding region of reflection symmetry and improves the prediction of the object surface. We use perceptual loss that considers the 3D shape encoder as a high-level 3D shape features extractor to differentiate between the original shape and the predicted shape. We employ a local perceptual loss which tries to differentiate the local shape and a global perceptual loss, which keeps the naturalness of the global 3D shape in the reconstruction (Figure 2).

### 2.1. Symmetry Prior Fusion

**Figure 3:**
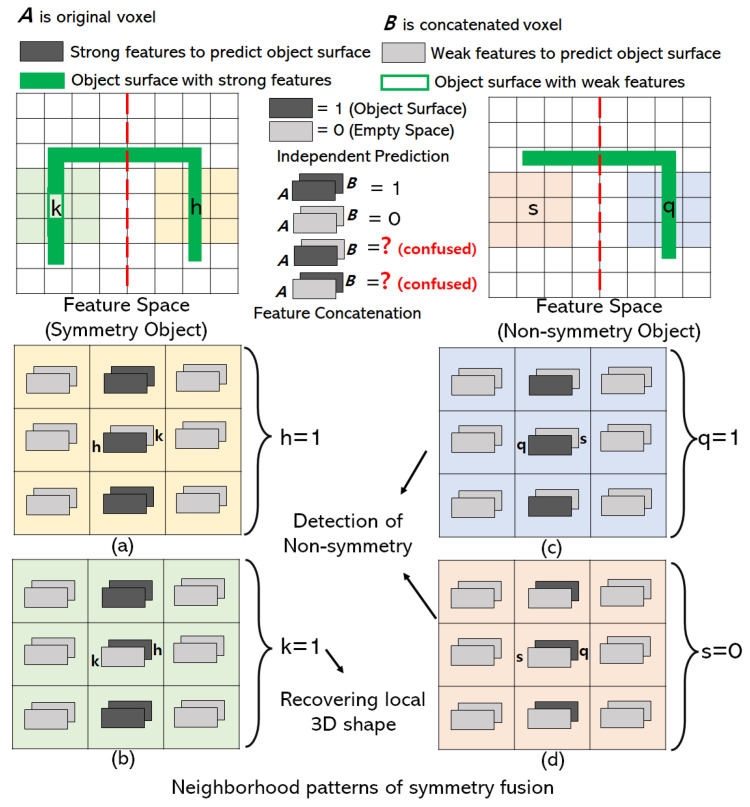
Symmetry fusion module recovers local shape and detects non-symmetry shapes. Network learns the neighborhood symmetry patterns for 3D reconstruction. The feature spaces of symmetry and non-symmetry object voxels in the symmetry fusion module are visualized as top view 2D grid for simplicity. The red dotted line is a symmetry plane in 2D. The solid green line represents the object surface with strong features. (**a**) Prediction of object voxel with weak features in symmetry structure. (**b**) Recovering object voxel with weak features. (**c**,**d**) Detection of Non-symmetry shapes.

The reflection symmetry plane is the x-y plane of object-centric coordinates where 3D objects are aligned with the canonical view. With symmetrical 3D objects, the reconstructed outputs of the 3D decoder are aligned with the canonical view as shown in Figure 1. The features of the 3D shape in the receptive field of the 3D decoder are aligned with the reconstructed output. So the local receptive field of the decoder is the symmetry in x-y plane as well. If ***B*** is a voxel located at the other side of the symmetry structure of voxel ***A***, then we share the feature of ***B*** inside the receptive field of 3D decoder with ***A*** by feature concatenation as shown in Figure 2. Concatenated features recover local shapes from symmetry prior considering the neighborhood patterns of symmetry fusion as shown in Figure 3. In the left of Figure 3, a symmetry object has a missing voxel *k* that has symmetry counterpart *h* reconstructed correctly. In this case, voxel *h* has strong features to be predicted as an object surface (Figure 3a) and the neighbor features of voxel *k* also support voxel *h* to be predicted as the object’s surface. On the other hand, voxel *k* has weak features and is going to be predicted as an empty surface. However, strong neighbor features from *h* influence *k* to be predicted as the object surface after symmetry fusion is applied as shown in Figure 3b.

As the symmetry features are explicitly added to the network, the symmetry fusion module does not affect the reconstruction of 3D shapes which are partially non-symmetry. In Figure 3c,d, we show that neighborhood patterns of symmetry fusion are able to find the non-symmetry shapes of an object. In Figure 3c, voxel *q* lies on non-symmetry structure of an object and weak neighbor features of *s* are not able to influence *q* to be predicted as empty space because neighbor features of *q* are strong. In Figure 3d, voxel *s* lies on the other side of the non-symmetry structure of an object and weak neighbor features of *s* influence *s* to be predicted as empty space even though neighbor features of *q* is strong. To include the neighborhood patterns, we add a symmetry fusion module prior to the prediction layer of the 3D decoder at a resolution of 16×16×16.

### 2.2. Reconstruction Loss

In the first step of training, the 3D shape encoder and 3D decoder are trained to get the latent representation of 3D shapes. Both networks are optimized with reconstruction loss $l_{rec}$, which is defined as binary cross-entropy (BCE) between the reconstructed object *p* and ground truth 3D object gt. The BCE loss calculates the mean of binary cross-entropy of each voxel between *p* and gt. More formally we define $l_{rec}$ as follows:

(1) $$l_{rec}\left(gt,p\right)=\frac{1}{N}\sum_{i=0}^{N}\left[gt_{i}\log\left(p_{i}\right)+\left(1-gt_{i}\right)\log\left(1-p_{i}\right)\right],$$

where *N* represents the total number of voxels. gt and *p* denote the ground truth voxel and the corresponding predicted occupancy respectively.

In the second step of training, only the 2D image encoder is trained with MSE loss between the latent features z(I) from the 2D image encoder and the latent features z(gt) from the 3D shape encoder as shown in Equation (2). Here, latent features z(gt) are considered as a ground truth of the corresponding 3D shape.

(2) $$l_{lat}\left(z\left(gt\right),z\left(I\right)\right)=\frac{1}{n}\sum_{i=0}^{n}{\left(z_{i}\left(gt\right)-z_{i}\left(I\right)\right)}^{2},$$

where *I* represents input images and *i* represents the index of latent features. *n* is the number of latent features. Both the 2D image encoder and the 3D shape encoder give an output of the same sized latent features.

In the final step of the training, we initialize the 2D image encoder from the second step and the 3D decoder from the first step. Finally, we train the 2D image encoder and the 3D decoder with both loss $l_{rec}$ and $l_{lat}$ along with perceptual loss $l_{per}$.

### 2.3. Perceptual Loss

It is a difficult task to transfer local details observed from a 2D encoder to a 3D decoder in a latent space. To provide local guidance, we propose local perceptual loss $l_{perL}$ between partial region *X* of gt and corresponding region $\hat{X}$ of *p*. We pass both *X* and $\hat{X}$ to a pretrained 3D shape encoder and get the respective latent representation of the partial regions $z_{i}\left(X\right)$ and $z_{i}\left(\hat{X}\right)$.

(3) $$l_{perL}\left(X,\hat{X}\right)=\frac{1}{N}\sum_{i}^{n}{\left(z_{i}\left(X\right)-z_{i}\left(\hat{X}\right)\right)}^{2}.$$

We apply a 3D voxel mask *M* to both gt and *p* to find X=gt⊙M and $\hat{X}=p⊙M$, where ⊙ represents element-wise multiplication. The size of the 3D bounding box *M* is randomly picked with a condition that within the bounding box of gt should contain the object surface.

In the case of 3D reconstruction from single view, the goal is to recover the 3D shape to visually match the perfect undistorted 3D shape. The single 2D view might have multiple counterpart shapes which are perceptually acceptable. Using only reconstruction loss, the output can have a mean shape problem for a counterpart. To solve the problem, we apply a global perceptual loss $L_{perG}$ which is defined as follows:

(4) $$l_{perG}\left(gt,p\right)=\frac{1}{N}\sum_{i}^{n}{\left(z_{i}\left(gt\right)-z_{i}\left(p\right)\right)}^{2},$$

where $z_{i}\left(gt\right)$ are latent features of the 3D ground truth shape gt and *i* is the index of latent features and *p* represents the predicted shape. Usually perceptual loss is calculated from the network that is trained for object classification. In our method, we use the same 3D shape encoder for perceptual loss calculation. Our perceptual loss includes both $l_{perL}$ and $l_{perG}$. We define the perceptual loss $l_{per}$ as follows:

(5) $$l_{per}=l_{perL}+l_{perG}.$$

### 2.4. Network Architecture

The 3D shape encoder takes 32 × 32 × 32 3D voxels as an input. The global latent features z(gt) are directly learned from the input voxel through the 3D shape encoder. The 3D shape encoder consists of four 3D convolution blocks. Each 3D convolution block contains 3D convolution, batch normalization, and ReLu activation. Four 3D convolution layers are configured with kernel sizes of (4,4,4,4), strides of (2,2,2,1) and channels of 128,256,512,128 respectively. Our 3D shape encoder produces 128 latent features.

After getting the global latent feature vector from the 3D shape encoder, we put z(gt) to the 3D decoder to reconstruct the respective 3D voxel from the latent space as shown in Figure 2. The 3D decoder is designed with four deconvolution blocks including transpose 3D convolution, batch normalization, and ReLu activation. Four transpose 3D convolutions have kernel sizes of (4,4,4,4), strides of (1,2,2,2) and channels of (512,256,256,1), respectively. We apply an element-wise logistic sigmoid function in the output layer for each voxel. Prior to the prediction layer, we add our symmetry fusion module as shown in Figure 2.

The 2D image encoder takes an RGB image *I* as input. Latent feature vector z(I) is extracted by the 2D image encoder from a single view RGB image to learn the respective z(gt). In our implementation, ResNet [49] is used for the 2D image encoder. The 2D image encoder gets a 224 × 224 image as input and extracts 128 latent features. In testing, we remove the 3D shape encoder and keep only the 2D image encoder with a 3D decoder.

## 3. Experimental Evaluation

In this section, we demonstrate extensive evaluations including an elaborated ablation study of the proposed method compared with state-of-the-art methods.

### 3.1. Data Sets

The ShapeNet dataset [47] is the collection of 3D object models based on WorldNet Taxonomy. In our experiment, we have used a subset of ShapeNet which consists of 44 K subjects of 13 major categories out of 55 categories. Following [7], we split 80 percent of the whole dataset into a training set and the rest of the dataset into a testing set. In the training, the output is voxels which are rendered from a 3D computer-aided design (CAD) model. The rendered voxels are aligned with the canonical view. We use the rendering method of [7], which consists of 24 random view images with a size of 137×137 for each 3D CAD model. As the synthetic rendered images have a transparent background, we consider a uniform color background to the image for qualitative results visualization. To evaluate the proposed method with real-world images, we use Pix3D dataset [48] for test. Pix3D provides real-world shape images and perfectly aligned CAD models. Pix3D dataset consists of 395 CAD models from nine categories. Each 3D object model is associated with multiple real-world object images. Following [48], we conduct our testing on 2894 unoccluded and untruncated images from the chair category.

### 3.2. Evaluation Metric

To evaluate the quality of our proposed method, we obtain an occupancy map with threshold t=0.3 [11] and employ intersection over union (IoU) as a evaluation metric. More formally,

(6) $$IoU=\frac{\sum_{i}^{N}I\left(gt_{i}>0\right)I\left(p_{i}>t\right)}{\sum_{i}^{N}I\left(\left(I\left(gt_{i}>0\right)+I\left(p_{i}>t\right)\right)>0\right)},$$

where $p_{i}$ and $gt_{i}$ are the predicted probabilities of being in object volume and ground truth respectively. I(.) denotes an indicator function and *t* is the threshold for voxel binarization. Higher IoU means better reconstruction performance. We calculate F-score based on [50].

(7) $$F-score\left(d\right)=\frac{2P(d)R(d)}{P(d)+R(d)},$$

where *d* is the distance threshold. P(d) and R(d) represent the precision and recall, respectively.

(8) $$P\left(d\right)=\frac{1}{k_{R}}\sum_{r\in R}\left[\min_{g\in G}∥g-r∥<d\right],$$

(9) $$R\left(d\right)=\frac{1}{k_{G}}\sum_{g\in G}\left[\min_{r\in R}∥r-g∥<d\right],$$

where G and R are point clouds from ground truth and prediction, respectively. *k* is the number of points in G and R. To generate a point cloud from voxel, first we apply marching cubes [51] to generate mesh of 3D surface. Then, we sample 8192 points from a generated mesh to calculate F-score between point cloud from prediction and ground-truth following [11]. Based on the suggestion of [50], we consider d=1% of the side length of the reconstructed volume which is more indicative to evaluate the prediction quality. Higher F-score indicates better reconstruction.

### 3.3. Training Protocol

In the first phase of training, we use 32 × 32 × 32 voxels as input and output for the 3D shape encoder and 3D decoder. We train the 3D shape encoder and decoder using the Adam optimization technique [52]. The initial learning rate is set to 0.001 and the total number of epochs is 150 in the first stage of training. In the second step, we use random view 224 × 224 RGB images as input to train the 2D image encoder where the output is the latent features of a length of 128. In this case, target latent features are the output of the 3D shape encoder taking respective 3D voxels as the input. We augment our training input images by applying random flip, random background, random color and light jittering following the method [11]. In the training phase, we use an Adam optimizer [52] with an initial learning rate of 0.001 divided by 2 after every 150 epochs up to 500 epochs. Finally, we fine-tune 2D image encoder and 3D decoder jointly feeding a random view of input image for 150 epochs with both reconstruction loss $l_{rec}+l_{lat}$ and perceptual loss $l_{per}$. In this step, we initialize the 3D decoder from the pretrained model of our first step training and the 3D image encoder from the pretrained model of our second step training.

### 3.4. Ablation Studies

We define a baseline model that does not have a 3D shape encoder and a symmetry fusion module compared to the proposed Sym3DNet. With the baseline, we directly train the 2D image encoder and the 3D decoder without a symmetry fusion module with only reconstruction loss $l_{rec}$. To show the effectiveness of 3D embedding, we add the 3D shape encoder to the baseline and apply training step 1 with $l_{rec}$ loss. Finally, we train the 2D image encoder with $l_{lat}$ and achieve a significant improvement in terms of IoU and F-score as shown in Table 1.

**Figure 4:**
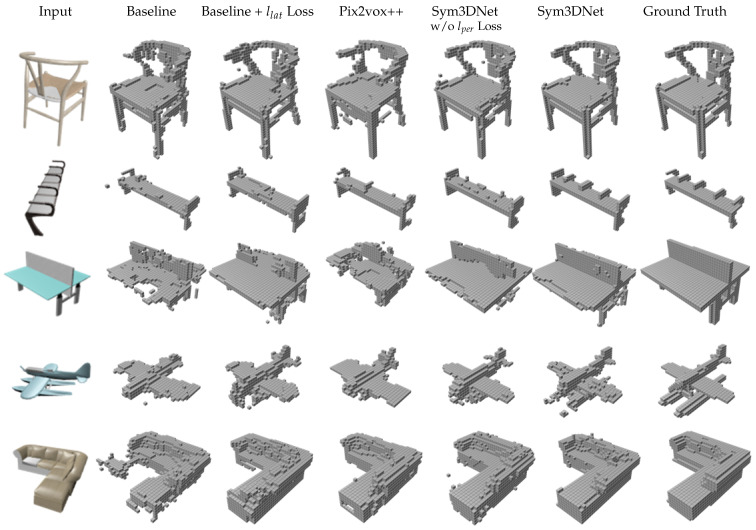
The effects of symmetry fusion, perceptual loss and 3D embedding in single view 3D reconstruction and comparison between baseline and state-of-the-art method Pix2vox++ [11]: Baseline network consists of 2D image encoder and 3D decoder without symmetry fusion module. We provide 3D embedding from 3D shape encoder for baseline (with $l_{lat}$) network. We also compare Sym3DNet without perceptual loss ($l_{per}$).

Sym3DNet without $l_{per}$ loss shows better reconstruction results than baseline in terms of IoU and F-scores (Table 1). Reconstruction results of Sym3DNet without $l_{per}$ loss are mostly symmetric as shown in Figure 4. As Baseline and Pix2vox++ [11] do not provide any symmetry guidance of an object, small structures of a 3D shape are reconstructed independently without having any influence of respective symmetry structures of the object. The reconstruction depends only on the latent features extracted from the 2D input image. If the 3D decoder fails to reconstruct any local surface from latent features, the symmetry fusion module in the decoder helps to reconstruct the local surface influenced by the counterpart features of the symmetry surface guided by the symmetry fusion module. Baseline, Pix2vox++ and Sym3DNet without $l_{per}$ loss suffer from recovering small local details in 3D reconstruction. Applying perceptual loss $l_{per}$ to our proposed network, we get improved 3D reconstruction results recovering local details as shown in Figure 4 such as the small details of a bench in the second example and the lower parts of float plane in the fourth example. The fifth example in Figure 4 has a partially non-symmetry structure. The proposed symmetry guidance keeps the structure of the non-symmetry part and affects only the symmetry part.

We observe in our experiment that a bigger size of the latent feature vector preserves both local and global details of a 3D object as shown in Table 2 for the 3D-3D auto-encoder. However, the bigger size of latent representation is difficult to learn from a single view RGB image due to occluded and missing part of an object. In Table 2, we show a comparison of the IoU and F-scores of our method with different lengths of latent features for both 3D-3D and 2D-3D. In this study, we use Resnet18 [49] for the 2D image encoder.

To provide a more detailed analysis of transforming single view 2D input image into 3D latent representation, we train the 2D image encoder with different backbone networks as shown in Table 3. When the 2D image encoder is deeper, it produces a 3D representation from a single 2D image better.

#### 3.4.1. Evaluation on ShapeNet Data Set

**Figure 5:**
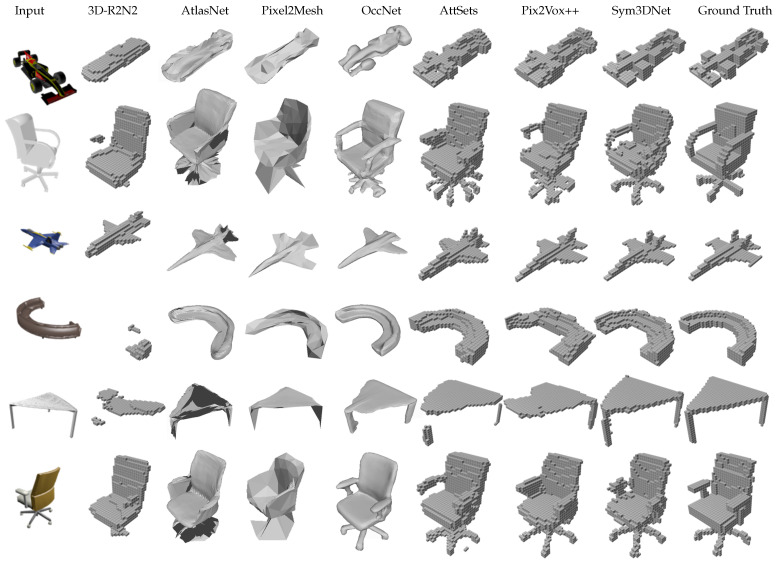
Examples of 3D shape reconstruction from single-view on synthetic dataset (ShapeNet): 32 × 32 × 32 is the resolution of reconstructed output volumes for voxel-based reconstruction methods.

We performed an experimental comparison of our method with several state-of-the-art methods including 3D-R2N2 [7], Matryoshaka [26], OGN [25], Pixel2Mesh [28], AtlasNet [53], IM-Net [54], OccNet [55], AttSets [10], Pix2Vox++ [11] on the ShapeNet test dataset. Table 4 and Table 5 show the IoU and F-Score of all methods. For fair comparison, we follow the same evaluation scheme described in [11]. The proposed method outperforms prior methods in both IoU and F-score that are illustrated in Table 4 and Table 5. Figure 5 shows single-view 3D reconstruction results, where we observe that the proposed method generates cleaner and more visually convincing 3D shapes than others. For example, the proposed method shows better reconstruction results with legs and handles of chairs, small details in the car and the tail of the plane, and so forth, as shown in Figure 5.

#### 3.4.2. Evaluation on Unseen Data

**Figure 6:**
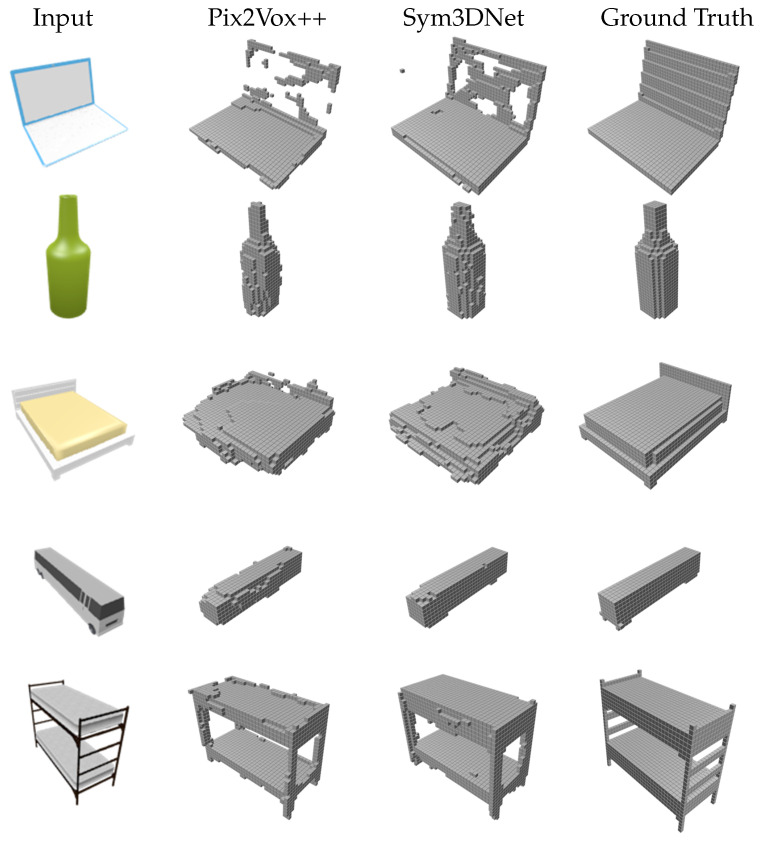
Single view reconstruction on unseen object from ShapeNet datasets.

We conduct an additional test to evaluate the generalization ability of our method to unseen shape reconstruction. For this experiment, we collect 3D shapes from the remaining 44 categories of the ShapeNetCore [47] dataset and render 2D views using the blender. For testing unseen shapes, we use a pretrained model. During the training, our model has never seen these shape categories. More specifically, we use 13 major categories of ShapeNet rendered by [56] for training and selecting shapes from the remaining categories for the test. In Figure 6, we demonstrate a visual comparison of our method with the state-of-the-art method [11]. We use their pretrained model [11] trained with the same data for the visualization of their test result on unseen shape reconstruction. Our result on an unseen class in Figure 6 shows that our model reasonably reconstructs the unseen 3D shape. This result indicates that the latent representation of a 3D shape preserves the structural information. Our 2D image encoder extracts the structural information from the 2D RGB image.

#### 3.4.3. Evaluation on Real-World Images

To evaluate the performance of our method for real-world single view reconstruction, we test our model on the Pix3D [48] dataset. We train our model on ShapeNet-chair and test on the chair category of the Pix3D dataset. For training, we render 20 random views with real-world background for each chair model of ShapeNet [47]. The background contains uniform color, long-dynamic-range scene, and randomly sampled indoor scene from SUN database [57]. In addition, we augment training data with a random color and jittering lighting. At first, we crop the input image with a bounding box and then rescale it according to the requirement of the network. We provide mean IoU and F-scores of the Pix3D dataset result in Table 6. The proposed method performs better than the state-of-the-art methods in terms of both IoU and F-scores.

**Figure 7:**
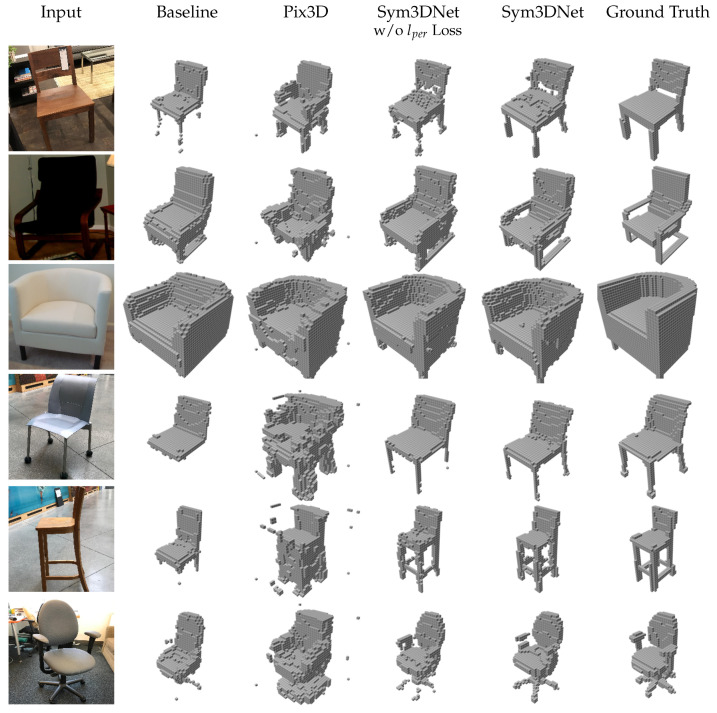
Three dimensional reconstruction from single view on the Pix3D testing set of chair category.

**Figure 8:**
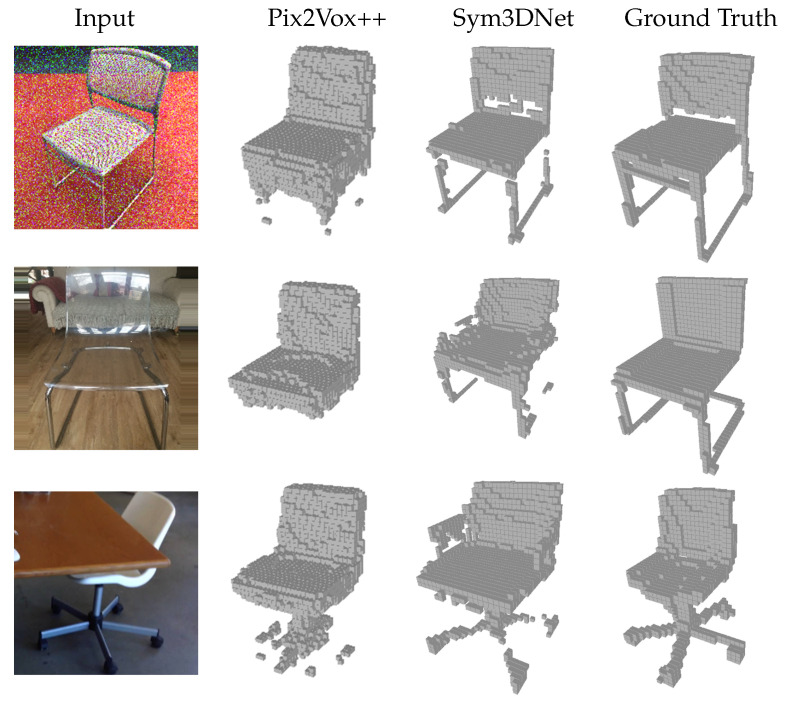
Single view reconstruction of proposed method on challenging situations such as noisy input, occluded and transparent object.

Figure 7 shows several real-world sample results showing that our method generates visually better and cleaner results. For the visualization of [48], we collect their testing result which contains the prediction probability and apply a threshold of 0.1 to create binary voxel grids. In Figure 8, our results have a promising reconstruction on challenging 2D inputs such as occlusion, transparent shape and noisy input.

#### 3.4.4. Space and Time Complexity

**Figure 9:**
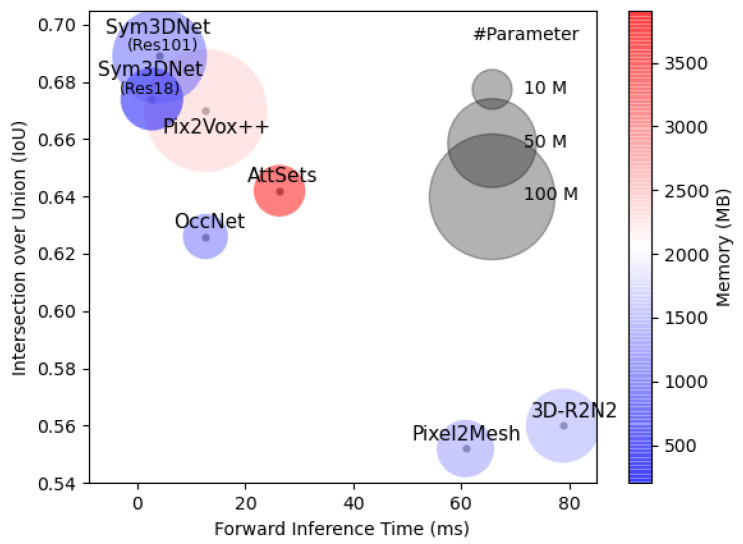
Intersection over Union (IoU), Model size, Number of Parameters and memory consumption of state-of-the-art methods for 3D shape reconstruction from single-view: The radius of the circle represents the size of corresponding models with respect to the number of parameters. Color of the circles represents the memory usage by the respective model (blue is lower and red is higher). In terms of inference time, IoU, and Memory usage, the proposed method outperforms all state-of-the-art methods.

For computational complexity, we compare the memory usage, inference time and number of parameters of the proposed method with several state-of-the-art methods as shown in Table 7 and Figure 9. For fair comparison, we collect all values of Table 7 from [11] and follow their scheme to obtain the values of the proposed method.

## 4. Conclusions

In this paper, we propose a symmetry 3D prior network for single-view 3D shape reconstruction. In our proposed method, we introduce a symmetry feature sharing scheme to infer the missing shapes from single-view and perceptual loss, which consists of both global and local terms. Global perceptual loss recovers the naturalness of the 3D shape and local perceptual loss recovers the detailed structures of a 3D shape. The proposed method outperforms state-of-the-art methods in 3D reconstruction in both quantitative and qualitative evaluations. Our method is computationally efficient compared to prior methods, considering both memory and time.

## Figures and Tables

**Table 1:** The effect of the symmetry fusion module and perceptual loss in our proposed network in terms of IoU and F-scores.

	3D Embedding	Symmetry Fusion	Perceptual Loss	IoU	F-Scores
Baseline				0.657	0.396
Baseline+$l_{lat}$ Loss	√			0.677	0.427
Sym3DNet w/o $l_{per}$ Loss	√	√		0.686	0.437
Sym3DNet	√	√	√	0.689	0.440

**Table 2:** Comparing IoU and F-scores of our method with different length of latent feature vector. We highlight the best scores in bold.

Lenth	3D-3D		2D-3D	
	IoU	F-Scores	IoU	F-Scores
64	0.847	0.601	0.670	0.417
128	0.873	0.640	**0.673**	**0.429**
256	0.891	0.658	0.666	0.422
512	**0.900**	**0.673**	0.662	0.421

**Table 3:** Comparing IoU Result with different backbone network for the 2D image encoder. We highlight the best scores in bold.

Backbone	IoU	F-Scores
VGG	0.593	0.362
ResNet18	0.673	0.429
ResNet50	0.683	0.437
ResNet101	**0.689**	**0.440**

**Table 4:** Comparison of 3D shape reconstruction from single view on ShapeNet13. We provide Intersection of Union (IoU) scores per category. We provide the mean IoU of each category. For the best category, we highlight the scores in bold.

Category	3D-R2N2	Matryoshka	OGN	Pixel2Mesh	AtlasNet	IM-Net	OccNet	AttSets	Pix2Vox++	Sym3DNet
	[7]	[26]	[25]	[28]	[53]	[54]	[55]	[10]	[11]	
airplane	0.513	0.647	0.587	0.508	0.493	0.702	0.532	0.594	0.674	**0.710**
bench	0.421	0.577	0.481	0.379	0.431	0.564	0.597	0.552	0.608	**0.656**
cabinet	0.716	0.776	0.729	0.732	0.257	0.680	0.674	0.783	0.799	**0.811**
car	0.798	0.850	0.828	0.670	0.282	0.756	0.671	0.844	0.858	**0.872**
chair	0.466	0.547	0.483	0.484	0.328	**0.644**	0.583	0.559	0.581	0.600
display	0.468	0.532	0.502	0.582	0.457	0.585	**0.651**	0.565	0.548	0.580
lamp	0.381	0.408	0.398	0.399	0.261	0.433	**0.474**	0.445	0.457	0.473
speaker	0.662	0.701	0.637	0.672	0.296	0.683	0.655	0.721	0.721	**0.723**
rifle	0.544	0.616	0.593	0.468	0.573	**0.723**	0.656	0.601	0.617	0.652
sofa	0.628	0.681	0.646	0.622	0.354	0.694	0.669	0.703	0.725	**0.740**
table	0.513	0.573	0.536	0.536	0.301	0.621	**0.659**	0.590	0.620	0.629
telephone	0.661	0.756	0.702	0.762	0.543	0.762	0.794	0.743	0.809	**0.814**
watercraft	0.513	0.591	0.632	0.471	0.355	0.607	0.579	0.601	0.603	**0.626**
Overall	0.560	0.635	0.596	0.552	0.352	0.659	0.626	0.642	0.670	**0.689**

**Table 5:** The comparison of 3D shape reconstruction from single view on ShapeNet13. We provide F-scores for each category. We use the marching cube algorithm for voxel reconstruction to triangular mesh output. For the best category, we highlight the scores in bold.

Category	3D-R2N2	Matryoshka	OGN	Pixel2Mesh	AtlasNet	IM-Net	OccNet	AttSets	Pix2Vox++	Sym3DNet
	[7]	[26]	[25]	[28]	[53]	[54]	[55]	[10]	[11]	
airplane	0.412	0.446	0.487	0.376	0.415	0.598	0.494	0.489	0.583	**0.596**
bench	0.345	0.424	0.364	0.313	0.439	0.361	0.318	0.406	0.478	**0.492**
cabinet	0.327	0.381	0.316	**0.450**	0.350	0.345	0.449	0.367	0.408	0.425
car	0.481	0.481	0.514	0.486	0.319	0.304	0.315	0.497	0.564	**0.574**
chair	0.238	0.302	0.226	0.386	0.406	**0.442**	0.365	0.334	0.309	0.302
display	0.227	0.400	0.215	0.319	0.451	0.466	**0.468**	0.310	0.296	0.313
lamp	0.267	0.276	0.249	0.219	0.217	**0.371**	0.361	0.315	0.315	0.324
speaker	0.231	0.279	0.225	0.190	0.199	0.200	0.249	0.211	0.152	**0.290**
rifle	0.521	0.514	0.541	0.340	0.405	0.407	0.219	0.524	0.574	**0.583**
sofa	0.274	0.326	0.290	0.343	0.337	0.354	0.324	0.334	0.377	**0.399**
table	0.340	0.374	0.352	0.502	0.371	0.461	**0.549**	0.419	0.406	0.385
telephone	0.504	0.598	0.528	0.485	0.545	0.423	0.273	0.469	**0.633**	0.613
watercraft	0.305	0.360	0.328	0.266	0.296	0.369	0.347	0.315	0.390	**0.410**
Overall	0.351	0.391	0.368	0.398	0.362	0.405	0.393	0.395	0.436	**0.440**

**Table 6:** Comparison of 3D reconstruction from single-view on a real-world dataset (Pix3D) at 32 × 32 × 32 resolution.

Network	IoU	F-Scores
ShapeHD [24]	0.284	-
Pix3D [48]	0.282	0.041
Pix2Vox++ [13]	0.292	0.068
FroDo [58]	0.325	-
Sym3DNet w/o $l_{per}$ Loss	0.325	0.147
Sym3DNet	**0.346**	**0.150**

**Table 7:** Comparison of our method in terms of memory, time inference, and number of parameters with state-of-the-art methods. The memory is calculated considering the backward computation with batch size of 1.

Methods	Pixel2Mesh	AtlasNet	IM-Net	OccNet	AttSets	3D-R2N2	Pix2Vox++	Sym3DNet	Sym3DNet
	[28]	[53]	[54]	[55]	[10]	[7]	[11]	(resnet18)	(resnet101)
Parameters (M)	21.36	45.06	55.45	13.43	17.71	35.97	96.31	25.93	57.46
Memory (MB)	1289	1293	3935	955	3911	1407	2411	158	892
Inference Time (ms)	60.78	38.47	10,886	12.61	26.32	78.86	10.64	2.68	4.11
